# Response of *Origanum dictamnus* L. (Cretan dittany) to Five Species of Root-Knot Nematodes (*Meloidogyne* spp.)

**DOI:** 10.2478/jofnem-2024-0037

**Published:** 2024-10-23

**Authors:** Emmanuel A. Tzortzakakis, Carolina Cantalapiedra-Navarrete, Pablo Castillo, Juan E. Palomares-Rius, Antonio Archidona-Yuste

**Affiliations:** Institute of Olive Tree, Subtropical Crops and Viticulture, Department of Viticulture, Vegetable Crops, Floriculture and Plant Protection, ELGO-DIMITRA, 32A Kastorias street, Mesa Katsabas, 71307, Heraklion, Crete, Greece; Institute for Sustainable Agriculture (IAS), CSIC, Avenida Menéndez Pidal s/n, 14004 Córdoba, Campus de Excelencia Internacional Agroalimentario, ceiA3, Spain.

**Keywords:** *M. javanica*, *M. incognita*, *M. arenaria*, *M. hapla*, *M. luci*, *Sideritis syriaca*, Crete

## Abstract

Cretan dittany (*Origanum dictamnus* L.) is an aromatic and medicinal plant, local endemic of the island of Crete, Greece, occurring naturally to high rocky mountain habitats. Due to its commercial interest, cultivation of this plant has been recently expanded. Since natural infestations by *Meloidogyne* spp. in field cultivated plants have not been found, its response to infection by *M. javanica*, *M. incognita*, *M. arenaria*, *M. hapla* and *M. luci* was investigated in pot experiments. In all experiments, roots of dittany plants exhibited neither galls nor egg masses in contrast to the roots of tomato plants used as controls. Therefore, Cretan dittany appears to be resistant to the five *Meloidogyne* species tested.

The cultivation of aromatic and medicinal plants has been recently expanded in Greece, both in traditional agricultural areas and in lands with thin soils that had been abandoned or had never been cultivated before. Root-knot nematodes (RKN), *Meloidogyne* spp., are among the most economically important nematodes in agriculture and have been recorded in several areas of Greece (Tzortzakakis et al., 2011). Considering infection of aromatic and medicinal plants in Greece by RKN, there are only two reports from the mainland country in cultivated balm (*Melissa officinalis* L.) and lavender (*Lavandula angustifolia* Mill) by *Meloidogyne arenaria* and by *M. hapla* respectively (Karanastasi et al., 2008; Gonçalves et al., 2020). In the island of Crete, infection by *M. javanica* and *M. incognita* has been reported in aloe (*Aloe vera* L.) and by *M. javanica* and *M. hapla* on cultivated Cretan mountain tea “malotira” (*Sideritis syriaca* L.) (Palomares-Rius et al., 2015; Tzortzakakis et al., 2019).

Cretan dittany (*Origanum dictamnus* L.) is an endemic plant of Crete, growing naturally to rocky mountain habitats at elevations above 500 meters. It is a short green-white, lanate shrub with a height up to 35 centimeters and stems ascending and rooting at their bases. Since ancient times, it has been considered as “panacea”, a type of drug for all types of human illness (Liolios et al., 2010). The most notable reference to its medicinal power came from Aristotle (4^th^ century BC), who noted that wounded wild goats of Mount Ida (Crete) consumed its aerial parts for the arrows to leave their bodies and of healing the wounds. Additionally, Galen, the famous Greek physician known as the “father of Pharmacy” (2^nd^ century AD), attributed to dittany healing, as well as anti-rheumatism properties (Liolios et al., 2010).

Dittany is a species of significant commercial interest since it is widely used for herbal teas and as a potential culinary ingredient (Krigas et al., 2015). Historically, it was collected from natural areas; however, today the plant material used comes exclusively from cultivated plants.

Plants of the Cretan mountain tea “malotira”, exhibiting symptoms of stunting and chlorosis, were found in a commercial crop in Arkalochori, Heraklion Province, Crete. The roots contained galls and egg masses, typical of RKN infection. Dittany plants were grown also closely to the nematode infected plants of “malotira” but did not indicate symptoms of nematode infection either on the upper part or galls in roots. Egg masses were picked from the “malotira” galled roots and used to inoculated tomatoes (*Solanum lycopersicum* L.) cv ACE grown in pots. After a 60 days period in a growth room at 22–25 °C and 16 h photoperiod, the plants were uprooted, the roots were washed and several nematode stages (female, male and second-stage juveniles (J2s) specimens) were used to identify the species by morphology and molecular markers. Specifically, mature females, males and J2 of *Meloidogyne* were selected to study the morphometrics, including stylet length, de Mann ratios, and perineal pattern, prepared and examined as described by Hartman and Sasser (1985). Females were also used for molecular identification as stated below. The nematode population was identified as *M. javanica* (designated thereafter as *M. javanica*-field). Egg masses were used to inoculate tomatoes cv. ACE to increase the nematode population, and the tomato hybrid Alliance F1 (Clause), containing the *Mi* gene, to investigate whether the field nematode population can overcome the resistance provided by this gene. This *M. javanica*-field population, was not able to reproduce on the nematode-resistant tomato hybrid.

Since the Cretan dittany plants were not found to be infected by RKN in the field, we investigated their response to the *M. javanica* - field population, found in “malotira” crop and to six additional populations of RKN representing five species present in Greece. These populations were maintained in pot cultures with tomatoes cv. ACE (*M. javanica*, *M. arenaria*, *M. hapla* and *M. luci*) and peppers cv. California Wonder (*M. incognita*), for long periods. These pots were kept in a growth room at 22–25 °C and 16-hour photoperiod. The seven populations used in the experiments were:

a) *M. javanica* - virulent on *Mi* gene of tomato, originating from an outdoor tomato crop in Crete and identified by isozyme phenotypes and a multiplex PCR assay (Tzortzakakis et al., 1999; 2005)
b) *M. incognita*-G, originating from a grapevine in Crete (unpublished) and identified using a multiplex PCR assay, which was carried out using species-specific primers Mi2F4/Mi1R1 for *M. incognita*
c) *M. incognita*-K, originating from an outdoor tomato crop in Kyparissia, Peloponissos and identified by isozyme phenotypes and a multiplex PCR assay (Tzortzakakis et al., 2005)
d) *M. arenaria*, originating from a balm crop from Thrace, North Greece and identified by isozyme phenotypes (Karanastasi et al., 2008)
e) *M. hapla*, originating from lavender crop from Kozani, North Greece and identified by isozyme phenotypes (Gonçalves et al., 2020)
f) *M. luci* originating from maize crop from Kavalla, North Greece and identified by isozyme phenotypes and sequence data (18S rDNA) (Conceição et al., 2012; Gonçalves et al., 2020) and
g) *M. javanica* - field which was stated before, originating from malotira crop in Crete and identified using species-specific primers Fjav/Rjav for *M. javanica*.

The populations a–f, which were maintained in pots were re-identified to species before being used in the experiments. *Meloidogyne javanica*, *M. incognita* and *M. arenaria* were identified using a multiplex PCR assay (Kiewnick et al. 2013), which was performed with species-specific primers (Kiewnick et al., 2013; Zijlstra et al., 2000). Specific bands for each species were detected: Far/Rar for *M. arenaria*, Mi2F4/Mi1R1 for *M. incognita* and Fjav/Rjav for *M. javanica*. The multiplex PCR cycling conditions were as follows: 95 °C for 15 minutes, 40 cycles at 94 °C for 30 seconds, 57 °C for 1 minute, and 68 °C for 2 minutes, with a final extension cycle of 68 °C for 9 minutes. PCR volumes were adapted to 20 μl for each reaction, and primer concentrations were as described in Kiewnick et al. (2013). On the contrary, for molecular identification of *M. hapla* and *M. luci,* the cytochrome oxidase subunit II (COII) gene region of mtDNA was amplified and sequenced with primers C2F3 and 1108 (Powers and Harris 1993). The obtained sequences were used in a BLAST search in the GenBank for their identification and matched (99.9%) with previous sequences deposited in NCBI. The molecular analyses of these populations including the multiplex PCR, as well as COII sequences, confirmed their identity and agreed with descriptions of *M. javanica, M. incognita*, *M. arenaria*, *M. hapla*, and *M. luci* (Subbotin et al., 2021), corroborating the previous findings regarding the identity of each nematode population.

Dittany plants were planted in 300 ml plastic pots filled with a commercial soil substrate (Humin Substrat, Klasmann-Deilmann GmbH, Germany; pH 6, organic matter 90%). Eggs of RKN were extracted from infected roots (Hussey and Baker 1973) and used for plant inoculation at a rate of 2,500 per plant. The plants grew in a growth room at 22–25°C and 16-hour photoperiod for 60 days. After this period, the plants were uprooted, their roots were washed, and the number of galls and visible egg masses were assessed. Tomato plants cv ACE were used as controls to prove the viability of nematode inoculum.

All experiments were conducted twice over time, with 5–12 replicates for dittany plants and 4–6 replicates for tomatoes. In all cases, roots of dittany plants did not have either galls or egg masses while roots of tomatoes were severely infected (Table 1, Fig. 1). Therefore, dittany appears to be resistant to the five evaluated RKN species.

**Figure 1:**
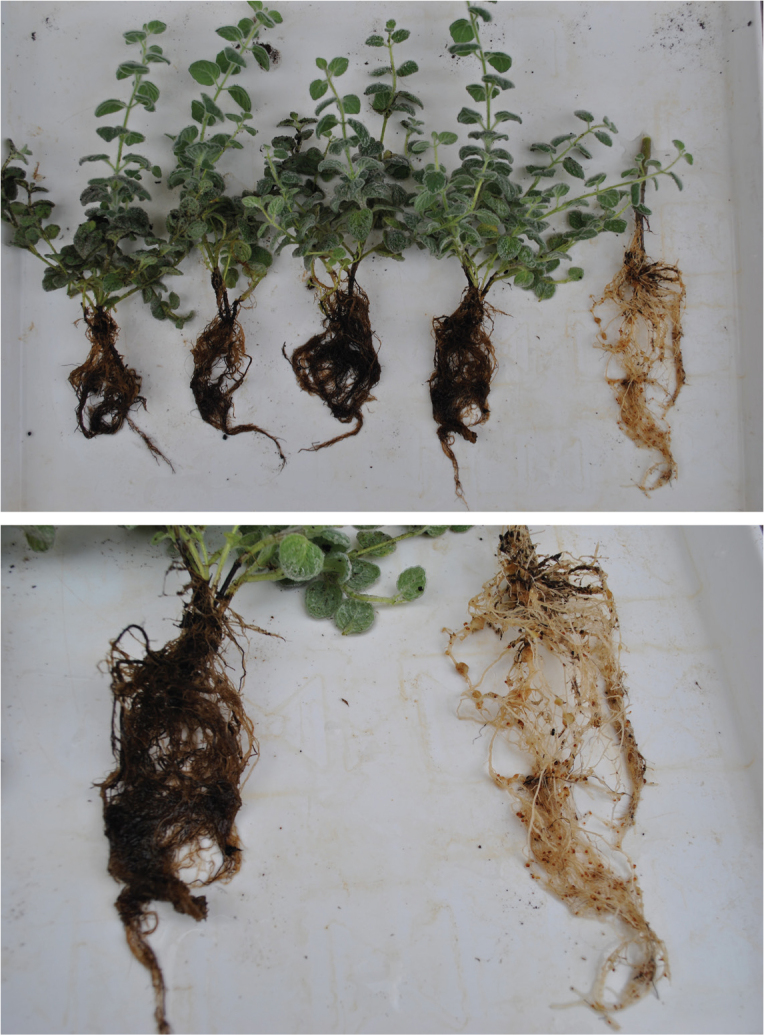
Roots of dittany plants (four in the left) and tomato (one in the right) after a two month period of infection with eggs of *M. javanica*.

**Table 1:** Number of galls and egg masses on roots of tomato and dittany plants inoculated with 2,500 eggs of *Meloidogyne* populations.

**Nematode population**	**Tomato cv. ACE**				**Dittany**			

	**Galls**		**Egg masses**		**Galls**		**Egg masses**	
	
	**Exp 1**	**Exp 2**	**Exp 1**	**Exp 2**	Exp 1*	Exp 2*	**Exp 1**	**Exp 2**
*M. javanica*-field	235	186	83	84	0 (12)	0 (6)	0	0
*M. javanica*-virulent	287	204	110	126	0 (9)	0 (6)	0	0
*M. incognita*-G	243	170	117	98	0 (5)	0 (5)	0	0
*M. incognita*-K	257	208	120	146	0 (10)	0 (5)	0	0
*M. arenaria*	265	196	79	110	0 (10)	0 (6)	0	0
*M. hapla*	178	130	98	76	0 (12)	0 (6)	0	0
*M. luci*	288	220	96	128	0 (12)	0 (10)	0	0

Each mean is average of 4–6 replicates for tomato and 5–12 replicates for dittany;

* the number of replicates for dittany in brackets.

Several polyphenolic components, including flavonoids and coumarins and essential oils with carvacrol as the major component, have been found in the aerial parts of the dittany plant. However, in one case of cultivated *O. dictamnus*, thymol (isomer of carvacrol) was found to be the dominant volatile constituent of its essential oil (Liolios et al., 2010). The essential oils of dittany have antimicrobial and insecticidal activity which could be attributed to their high content of carvacrol and its isomer thymol (Liolios et al., 2010; Krigas et al., 2015). Carvacrol and thymol, when used as pure commercial formulations, have been found effective against *M. javanica* (Nasiou and Giannakou, 2017; 2023). Since these compounds have been isolated from leaves of dittany and not from roots, it is not known whether the nature of resistance to RKN may be associated with their presence in the plant, necessitating further investigation.
